# State-Space Modelling of the Drivers of Movement Behaviour in Sympatric Species

**DOI:** 10.1371/journal.pone.0142707

**Published:** 2015-11-18

**Authors:** F. J. Pérez-Barbería, M. Small, R. J. Hooper, A. Aldezabal, R. Soriguer-Escofet, G. S. Bakken, I. J. Gordon

**Affiliations:** 1 Ungulate Research Unit, CRCP, University of Córdoba, Córdoba, Spain; 2 Grupo PAIDI RNM118, Estación Biológica de Doñana, CSIC, Sevilla, 41092, Spain; 3 James Hutton Institute, Craigiebuckler, Aberdeen, AB15 8QH, Scotland, United Kingdom; 4 School of Mathematics and Statistics, The University of Western Australia, Crawley, WA, Australia, 6009; 5 Landare Biologia eta Ekologia Saila, Zientzia eta Teknologia Fakultatea, Euskal Herriko Unibertsitatea (UPV-EHU), 644 p.k., 48080 Bilbo (Bizkaia), Euskal Herria, Spain; 6 Estación Biológica de Doñana, C.S.I.C. Apartado 1056, Sevilla, 41013, Spain; 7 Department of Biology, Indiana State University, 200 North Seventh Street, Terre Haute, Indiana, United States of America, 47809–1902; 8 Deputy Vice Chancellor, Tropical Environments & Societies, James Cook University, Townsville, Qld 4811, Australia; Centre for Cellular and Molecular Biology, INDIA

## Abstract

Understanding animal movement behaviour is key to furthering our knowledge on intra- and inter-specific competition, group cohesion, energy expenditure, habitat use, the spread of zoonotic diseases or species management. We used a radial basis function surface approximation subject to minimum description length constraint to uncover the state-space dynamical systems from time series data. This approximation allowed us to infer structure from a mathematical model of the movement behaviour of sheep and red deer, and the effect of density, thermal stress and vegetation type. Animal movement was recorded using GPS collars deployed in sheep and deer grazing a large experimental plot in winter and summer. Information on the thermal stress to which animals were exposed was estimated using the power consumption of mechanical heated models and meteorological records of a network of stations in the plot. Thermal stress was higher in deer than in sheep, with less differences between species in summer. Deer travelled more distance than sheep, and both species travelled more in summer than in winter; deer travel distance showed less seasonal differences than sheep. Animal movement was better predicted in deer than in sheep and in winter than in summer; both species showed a swarming behaviour in group cohesion, stronger in deer. At shorter separation distances swarming repulsion was stronger between species than within species. At longer separation distances inter-specific attraction was weaker than intra-specific; there was a positive density-dependent effect on swarming, and stronger in deer than in sheep. There was not clear evidence which species attracted or repelled the other; attraction between deer at long separation distances was stronger when the model accounted for thermal stress, but in general the dynamic movement behaviour was hardly affected by the thermal stress. Vegetation type affected intra-species interactions but had little effect on inter-species interactions. Our modelling approach is useful in interpreting animal interactions, in order to unravel complex cooperative or competitive behaviours, and to the best of our knowledge is the first modelling attempt to make predictions of multi-species animal movement under different habitat mosaics and abiotic environmental conditions.

## Introduction

Ungulates are a key component of many ecosystems across the world, and play a fundamental role in ecosystem dynamics, for example through acting as a trophic link between primary producers and omnivores, as ecosystem engineers by their grazing and browsing impact, as a resource for humans in the form of hunting food, domesticated animal stock, source of fibre, transportation and recreation, among others [1–3].

In developed countries the drastic reduction in extensive livestock farming and abandonment of traditional management practices has affected the condition of many habitats, some of which are hot-spots of biodiversity [4], and has been the cause of the rapid increase in population density and spatial distribution of some wild ungulate species [5]. This has had a negative effect on the rural economy [6,7], and altered the competitive interactions between wild species [8–10]. There is growing interest in maintaining extensive traditional livestock production, together with viable wild ungulate species populations, as (i) there is evidence that intermediate levels of grazing intensity favour biodiversity of plants and assemblages of insects and vertebrates [11–13]; and (ii) the revenue of sustainable hunting and associate tourism industry benefit deprived rural areas [14,15]. However, there is also evidence of disease transmission between wild and domestic species, with devastating effects on populations of both types of herbivores [16–18].

Understanding animal movement is key to furthering our knowledge of energy expenditure, habitat use, spread of zoonotic diseases and species management [19–21]. However, animal movement is intrinsically stochastic and often affected by observation error, which make dynamic models of movement a major challenge for behavioural ecology [22].

There are three main factors involved in determining the spatial distribution of an individual, its preference for particular habitats, its interactions with other individuals of the same or different species (competition-facilitation-predation), and the effect that weather has on energy expenditure [21,23–30]. The effect of weather variables on population dynamics modelling and its impacts on body mass and condition, reproduction and mortality are well studied [31–33]. However, because of the difficultly to capture the animal response to rapid changes in weather conditions in real time, little is known about the effect of microclimatic conditions, or any proxy of thermal stress, on animal movement [26,34–36].

Species interactions, together with density-dependent habitat selection, are key processes underlying population ecology, and are important in supporting evidence-based management of species assemblages, biodiversity and the ecosystem services [37–41]. For example, in ungulates, there is evidence that increasing density can have negative effects on habitat, body condition and population density of a competing species [8–10]. In terms of habitat impact, differential defoliation of vegetation between habitats by herbivores affects spatial variation in plant growth [42], nutrient cycling [43] and vegetation structure [44], which indirectly affects habitat for plant and animals [45] and landscape aesthetics [3].

There is a plethora of studies that assess habitat use in ungulates using static models of observations at a particular time across different habitats [46–49]. Their conclusions are limited to the detection of positive or negative co-occurrence of two or more animals nearby, which provides no information on the direction of the interactions, nor any information about the nature of the competition. More realistic approaches compare the actual spatial distribution against a randomisation of the underlying habitats, or against a randomisation of the positions of the observations retaining or not the point pattern of the observations [50–52]}. A dynamic approach, the state–space model, enables the separation of the real biological signals from the observation error by coupling a statistical model of the observation method with a model of the movement dynamics [22].

The aim of this study is to build a state-space model to predict individual movement of animals of two species (sheep and red deer) based on (1) relative position of other members of the same specie; (2) relative position of members of the other specie; (3) thermal stress; and (4) land cover (i.e. vegetation types). We hypothesised that sheep and deer movement will show swarming group cohesion (H1): repulsion at short distances, both conspecifics and between species, as a consequence of proximity avoidance between individuals by competition for food resources; attraction at medium ranges, as individuals and both species use the same resources which are patchily distributed; and alignment of velocities within species to maintain group bonds [41,53–55]; (H2): vegetation types which are preferred by both species will increase aggregation and will reduce the swarming effect in animal movement (i.e. weakening inter-species repulsion forces); (H3) as there are significant differences in thermal insulation between species (i.e. sheep are better insulated than deer)[56] thermal stress in harsh weather (winter vs. summer) will affect the structure of the inter-species interactions.

## Methods

### Study area and animals

**Fig 1:**
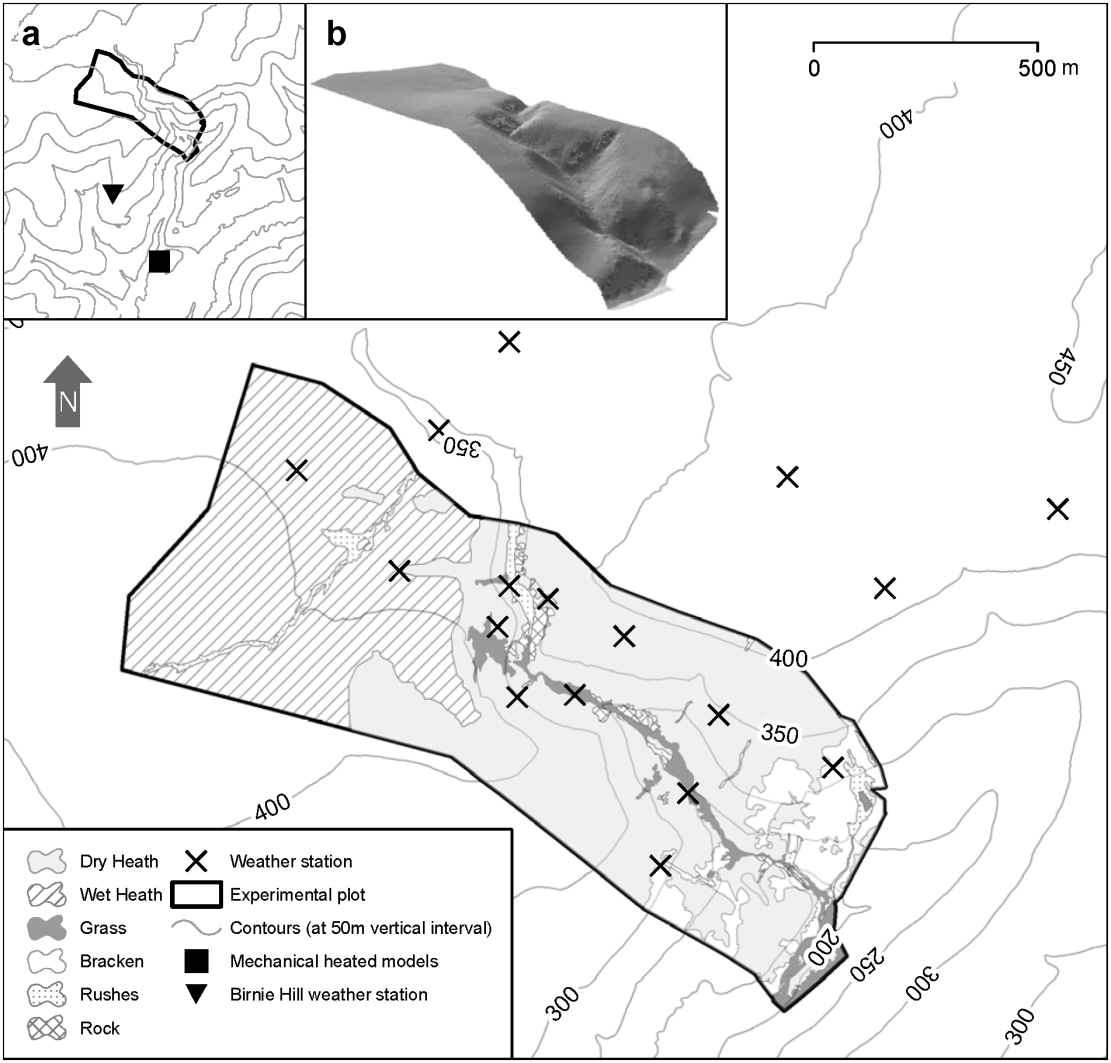
Experimental plot, vegetation types and situation of the meteorological stations and heated mechanical models used in this study. Main plot: crosses mark the positions of the meteorological stations used to create the surfaces of meteorological variables. Inset (a) indicates the position of the experimental plot and the near-by facilities used in this study, the plot where the mechanical heated models were deployed (solid square) and the meteorological station of Birnie Hill (solid inverted triangle). Inset (b) grayscale three-dimensional image representation of the experimental plot (see Methods for more details).

The experiment was carried out at the James Hutton Institute’s Glensaugh Experimental Field Station in Auchenblae (Aberdeenshire), North East Scotland (56° 29' 23.366" N, 2° 54' 41.273" W). We used a stock-proof fenced plot of 1.04 km^2^ located in a moorland area, (altitude: *mean* = 354.4 m, *min-max* = 178m-432m, *sd* = 62.14; slope: *mean* = 16.5°, *min-max* = 0°-49°, *sd* = 12.4, Fig 1). The plot was dominated by a mosaic of six vegetation types: bracken, dry heath, wet heath, grass, rock and rushes (S1 Table). The vegetation map was initially created by delineating areas of different vegetation types identified on aerial photography (OS MasterMap Imagery Layer, resolution: 25 cm; flown on 10/04/2003; accuracy, root mean square error: 1.1 m). Images were viewed on ArcMap 9.2 [57], and vegetation polygons were created using heads-up digitizing. Vegetation patches which were not identified from the imagery, but were identified from field observations, were mapped using a Trimble GeoXH handheld GPS.

We studied sheep (*Ovis aries*; Scottish Blackface breed) and farming red deer (*Cervus elaphus*), two species that have extensively grazed the Scottish glens, hills and moorland for at least 200 years [58,59]. Scotland has the largest population of wild red deer in Europe—a conservative estimate of 300,000 in 2004 [60]—and had around 7 million sheep in 2007, many of which are pastured extensively in the hills where both species share the same habitats (http://www.sac.ac.uk/mainrep/pdfs/retreatreport.pdf). In the last five decades there has been a concern that overgrazing imposes a threat to the biodiversity of these areas, particularly those dominated by heather moorland and blanket bog [61,62]. Scotland holds the majority of the global distribution of these habitats, which have been designated as Special Areas of Conservation under the EU habitats Directive (www.moorlandscotland.org.uk).

Between mid-June 2007 and December of 2008, 20 red deer adult hinds (age = 5–13 yr, *mean* = 10 yr, *mean* weight = 92.2 kg) and 60 ewes (age = 3yr, *mean* weight = 46.6 kg) grazed the plot. No males or calves/lambs were included in the study to simplify behavioural variation. Sheep and hinds were drawn from the field station animal stock kept in hill farming conditions. During this period human disturbance was reduced to the minimum husbandry required to ensure the welfare of the animals. Animals were not fed or supplemented throughout the duration of the study. The density of sheep in the plot was close to the maximum densities of hill sheep in the Scottish highlands [63], while the deer density was near the maximum for Scottish wild red deer recorded by the Deer Commission for Scotland between 1961–2004 [52,64]. Between mid-June 2007 and the end of December 2007 the animals were familiarised with the plot and records did not start until January 2008.

Seventeen hinds and 23 ewes were fitted with GPS collars (LOTEK GPS3300). Collars weighed 330 g, which was between 0.4% and 0.7% of the winter average body mass of the hinds and sheep, respectively (0.5% and 0.9% of the lightest hind and sheep). The GPS collars were programmed to record one location fix per hour, on the hour, throughout the study period. Location data from the collars were downloaded at the end of the experiment and all records were differentially corrected to improve accuracy. Trials undertaken in our plot indicated that 95% of the differentially corrected fixes were within a 14 m radius of their actual position. No adverse effect of the collars was detected across the experiment, other than some loss of fur around the collar strap in the hinds. Animals were gathered every 6 months to download the information of the GPS collars, change the collars’ batteries, and the animals received the standard farming prevention veterinary treatment. After the experiment the collars were removed and the animals were returned to the main flock. The study did not involve endangered or protected species, and GPS collars deployment on sheep or farming red deer do not required any special permission in UK (data set available S1 Data).

### Microclimate data

We used hourly information on wind speed, air temperature, relative humidity, rainfall and incident solar radiation recorded by 17 portable meteorological stations deployed across the experimental plot to produce spatial surfaces of each variable at a resolution of 10 m x 10 m (see Microclimate data in S1 Text for details). During the study air temperature ranged between -19.9 and +29.8°C (mean = 6.4°C, 1^st^ – 3^rd^ quartile = 1.6–10.8°C) and wind speed varied between 0—110 km h^-1^ (mean = 18.4 km h^-1^, 1^st^ – 3^rd^ quartile = 5.8—28.8 km h^-1^).

### Thermal stress and heated mechanical models

The energy budget of an animal can be modelled using an equation where heat produced by metabolism and heat absorbed by the body from radiation minus heat loss by convection, radiation and conduction, equals zero [29,65]. However, the experimental determination of the parameters of this equation at a small spatial and temporal scale were intractable in our experiment [29,35]. Therefore we determined the electrical consumption of heated mechanical models of deer and sheep as a proxy of comparative thermal stress, not an estimate of actual metabolic demands [66,67]. Two heated models, 1 sheep and 1 red deer, were deployed in the hill exposed to the same weather conditions experienced by the animals (Fig 1), an internal logger recorded electric consumption and the explanatory variables air temperature, wind speed, solar radiation, relative air humidity and rainfall in real time (see Thermal stress and heated mechanical models in S1 Text for the constructions of the heated mechanical models).

### Statistical analysis

#### Relationship between the thermal stress index and microclimate variables

Meteorological variables (i.e. air temperature, wind speed, solar radiation, relative air humidity and rainfall) strongly affect the animal's energy budget and hence their metabolic demands [29]. While animals may have specific behavioural responses to different microclimate factors which have significant thermoregulatory consequences (Bakken, 1981), we simplified the microclimate analysis by condensing all variables into the net rate of energy consumption (metabolic rate) [68]. Rather than construct an analytic heat transfer model predicting metabolic rate (Porter and Gates, 1969), we related the heater power of the mechanical models of sheep and deer to the meteorology variables at the heated models’ meteorology site using the conceptual approach of operative environmental temperature [65,69] (see Thermal stress index in S1 Text).

The information of the 17 meteorological stations was kriged (see Microclimate parameters in S1 Text) and then used as input data for the GAM model predicting hourly electrical consumption by the heated mechanical models to generate the final spatial surface of heater power across the study area at a resolution of 100 m^2^. This microclimate index will be referred to as 'thermal stress’ hereinafter.

#### Daily distance travelled

To describe the average daily distance travelled (in m) by sheep and deer in two thermal stress contrasting seasons (summer: April to November; winter: December and January to March) we fitted a linear mixed-effects model with species and season and their interaction as fixed effects and animal identity as random effect, using R v3.1.2 software (R Development Core Team 2012), lme4 v1.1–7 [70] and lmerTest v2.0–20 [71] packages. Normality and homoscedasticity were verified [72] and inspection of plots of fitted values against residuals did not reveal any outliers. As in linear mixed-effects models, determining the correct value of degrees of freedom in the estimate of the coefficients is meaningless [72,73], as an alternative we used Satterthwaite’s degrees of freedom approximation [71]. We plotted the predicted means of the model (least square means), their corresponding standard errors and 0.95 confident intervals, and pairwise comparisons between predicted means with p-values after Bonferroni correction [74]. Graphics were conducted using ggplot2 v1.0.1 R package [75].

#### Modelling deterministic dynamics and interactions

The fundamental idea underpinning the state-space model is that the deterministic behaviour of the system can be predicted from sufficient knowledge of the current state. If the current positions of the animals are known, and the state of the environment is adequately described, then one can predict future behaviour. Chaos places fundamental limits on how far into the future one can predict, but prediction is possible, in principle, in the short term. Typical ecological systems have a strong component of noise. Essentially, this noise blurs the state and makes prediction difficult. The role of the state-space model is to use the best-guess of the current state, contaminated by noise, to make an estimate of future behaviour. If the state-space model is accurate then that estimate will be the expected mean behaviour of the actual system. One can then use this state-space prediction of future behaviour to build a dynamic model. Simulation from the dynamic model can then be made by adding a typical random realisation of system noise to the state and then iterating the process–predict the future state, add noise, predict the next state, add more noise and so on. An ensemble of such predictions provides an estimate of potential future behaviours [76].

We sought to construct mathematical expressions for the strength of intra- and inter-specific interactions. This can be achieved by either (1) a statistical fit to the animal locations; or (2) via inferring structure, from a mathematical model, of the interaction and dynamical behaviour of the animal’s movement. The first approach is primarily operating under assumptions of stochastic interaction [for example, [71,72]], while the second seeks to unveil deterministic dynamics [77,78]. As our objective was a description of deterministic interaction behaviour between animals, we choose the second approach.

Our modelling approach builds on sophisticated radial basis modelling algorithms and minimum description length model selection ideas (S2 Text) that have been developed for understanding dynamic systems from time series [77,78] and have been applied to inter-specific interactions in ungulates [41].

At each instant in time we constructed interaction vectors for each animal. In Eq 1 si(t) denotes the two-dimensional position of sheep-i at time t, and, similarly dj(t) denotes the position of deer j at time t. The interaction vector for sheep i is:

$$\text{v}\left({\text{s}}_{\text{i}}\text{(t)}\right){\text{=(s}}_{\text{i}}{\text{-s}}_{\text{k}\left(\text{1}\right)}{\text{,s}}_{\text{i}}{\text{-s}}_{\text{k}\left(\text{2}\right)}{\text{,…,s}}_{\text{i}}{\text{-s}}_{\text{k}\left(\text{m}\right)}{\text{;s}}_{\text{i}}{\text{-d}}_{\text{h}\left(\text{1}\right)}{\text{,s}}_{\text{i}}{\text{-d}}_{\text{h}\left(\text{2}\right)}{\text{ ,…s}}_{\text{i}}{\text{-d}}_{\text{h}\left(\text{n}\right)}\text{)}$$ (1)

where k(i) is the i-th nearest neighbouring sheep and, similarly h(j) is the j-th closest deer. For succinctness, we dropped the dependence of t throughout the right-hand-side of Eq 1. Expressions of the form (1) provide a snapshot of the relative position of the m closest sheep to a target sheep and the n closest deer, of all sheep and deer individuals [41].

As we were also interested in how vegetation type and thermal stress influenced species interaction, in addition to the vector v(si(t)), we considered additional information on these factors to predict the future positions of individuals. These additional parameters are properties of the location of sheep i at time t, we denote these as w = f(v).

We employed the deterministic model scheme developed by Judd and Mees [77] and Small and Tse [78] to construct a function F(v(si(t)), f(v)) capable of predicting the updated position of sheep i at time t:

$$\text{F}\left(\text{v}\left({\text{s}}_{\text{i}}\left(\text{t}\right)\right)\text{;f(v)}\right)\approx {\text{s}}_{\text{i}}\left(\text{t+1}\right){\text{+e}}_{\text{i,t}}$$ (2)

where $\text{model error= }\Sigma_{\text{i},\text{t}}{\text{e}}_{\text{i},\text{t}}{}^{2}$ is minimised. Clearly, minimising the model error can be trivial if F is complicated enough (i.e. if it has enough parameters which may be tuned). An over-complex model will fit the training data better, but will not be able to generalise to new data.

To minimize error while limiting the complexity of the model we required two key components [77,78]. The first component was the radial basis function structure we implemented to give F shape (S2 Text). This choice was somewhat arbitrary but provided us with the ability to fit a functional surface for F. The second component was the minimum description length model selection criteria. Essentially, one could construct many different functions F that perform as required, and those with more model parameters will naturally perform better. The minimum description length principal provides a cost for those additional parameters. The procedure was based on the assumption that the most compact description was best (S2 Text).

Once we obtain a deterministic mathematical description F of the model dynamics for a given particular sheep i, we computed vectors of the form Eq (1) for all sheep and all times and built a single predictive model using Eq (2). Model predictions from this model were equally good (that is, the magnitude of the residual of the model prediction error showed no animal specific variation and the residuals themselves exhibited no inter-animal bias) when employed to predict future positions of all sheep.

As mentioned above the positions of all animals were vectors in two dimension and hence the function F mapped 2(m+n) dimensional points into 2 dimensions. However, the coordinate system we employed was arbitrary and we assumed that it did not affect our results. Hence we built separate functions Fx and Fy to predict the x-component of motion and the y-component of motion. Assuming coordinate independence we also constrained the model to be equivalent after rotation Fx = R Fy where R is the 2-by-2 matrix performing anticlockwise rotation through 90-degrees. Hence by applying R and R^-1^ to the data in the interaction vectors and the right-hand-side of Eq (2) we only needed to build a single model F and construct:

$$\text{F}\left(\text{v}\left({\text{s}}_{\text{i}}\left(\text{t}\right)\right)\text{;f(v)}\right)\text{= }\left[\begin{array}{c} \text{f}\left(\text{v}\left({\text{s}}_{\text{i}}\left(\text{t}\right)\right)\text{;f(v)}\right)\\ \text{0} \end{array}\right]\text{+R}\left[\begin{array}{c} \text{f}\left({\text{R}}^{\text{-1}}\text{v}\left({\text{s}}_{\text{i}}\left(\text{t}\right)\right){\text{;R}}^{\text{-1}}\text{f(v)}\right)\\ \text{0} \end{array}\right]\text{.}$$ (3)

Not only did this ensure radial symmetry in our interaction rules, but it also minimised the computational burden involved in the model.

With these deterministic models of interaction—F for the sheep and G for the deer (the model G is created in exactly the same manner as F, except the interchange of the role of sheep and deer in the preceding description) we constructed a series of interaction diagrams that expressed the force of attraction or separation between a sheep (deer) and the neighbouring n and m other sheep (deer) and deer (sheep). These diagrams express the deterministic dynamical behaviour of the model deduced from the original field data. The following provides a precise description of the underlying calculation.

We fixed m and n, using all individuals, and built a model F of the general form Eq (3). Then, using the field data that was used to build the model, we compute

$${\text{Δs}}_{\text{i}}\left(\text{t}\right)\text{= }\Sigma_{\text{l}=1}^{\text{n}\prime }\left\|{\text{s}}_{\text{i}}{\text{(t)-s}}_{\text{k}\left(\text{l}\right)}\text{(t)}\right\|$$ (4)

the mean separation between a sheep an its n’ ≤ n nearest neighbours, and,

$${\text{p}}_{\text{i}}\left(\text{t}\right)\text{= }\Sigma_{\text{l}=1}^{\text{n}\prime }\left\|\text{F}\left(\text{v}\left({\text{s}}_{\text{i}}\left(\text{t}\right)\right)\text{;f(v)}\right){\text{-s}}_{\text{k}\left(\text{l}\right)}\text{(t)}\right\|$$ (5)

the predicted separation (i.e. the model prediction of how far apart a sheep and its n’ nearest neighbour will be). Lastly, we created a scatter plot of Δsi(t) against pi(t)-Δsi(t) as a measure of the predicted change in separation between a sheep and its n’ nearest neighbours as a function of the distance to its neighbours. To investigate the effect of various environmental drivers on animal interaction we then modified the addition input vector w to include information about the vegetation type, thermal stress or seasonal change. Incorporating these factors generated new sets of interaction graphs and we can determine whether there were any significant effects by comparing interactions curves.

Following the procedure described above we arrived at the estimate of the strength of interaction among sheep, between sheep and deer, among deer, and between deer and sheep. This produces a one dimensional histogram at each distance. This histogram was then fitted with a Gaussian kernel smoothing function to produce a probability density function that was used to create plots that depicted the median of the histogram, which were the output of the model that we present in Results (were negative values represent attraction between individuals and positive values repulsion). Finally, we sought to assess the effect of animal density in the movement and interactions in sheep and deer. In the context of the dynamic spatial spread of animals of each specie (i.e. the density of the flock), we actually refer to as the average inter-animal distances. For a particular spatial distribution (a fixed “snap-shot” in time) we computed the average distance to the k–th nearest neighbours of each animal. That is, a function D(k) that says, on average, a circle of diameter less than D(k) (centered on a particular animal) will be expected to contain only (k-1) other individuals.

## Results

### Heated mechanical models

GAMs on the microclimate variables explained 84.4% and 91.1% of variance in electrical consumption to maintain a core temperature of 39.7°C of sheep and deer heated models, respectively (S1 and S2 Figs and S2 Table). All the predictors were highly significant in both species (S2 Table). The deer mechanical model responded to changes in weather conditions faster than the sheep model due to the lower insulation power of its hide. This was because, given that both models had similar heat capacity, the time constant is mass × specific heat / thermal conductance (i.e. large values mean a slower response to changes in thermal environment). This might explain why the deer model explained more variance than the sheep model. The smooth functions of rainfall and humidity were somewhat flat in comparison to the other predictors, while the smooth functions of air temperature and wind speed had the strongest response in both heated mechanical models (S1 and S2 Figs).

### Thermal stress

**Fig 2:**
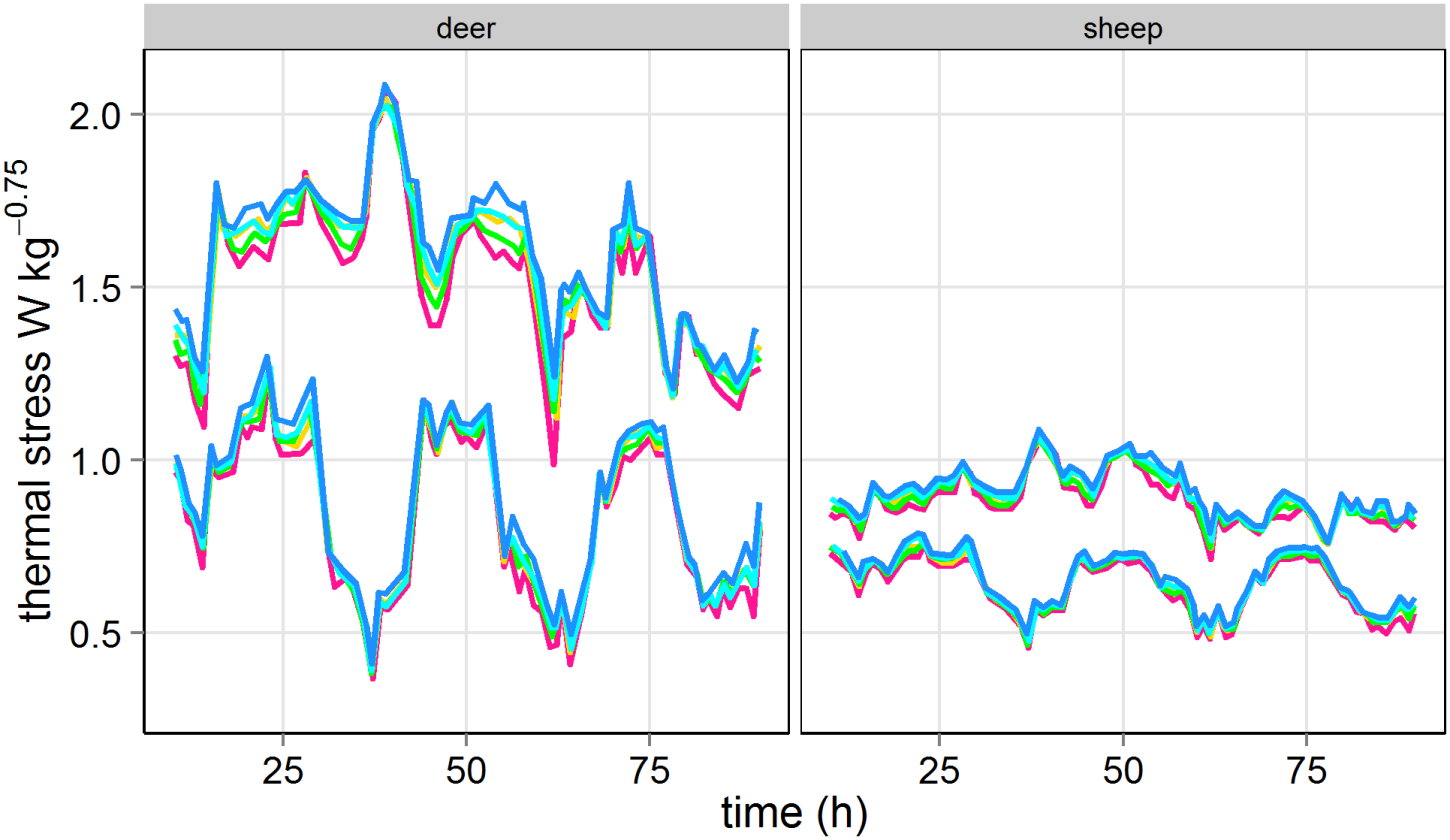
Winter and summer thermal stress in different vegetation types (bracken: pink; dry heath: yellow; grass: green, rushes: light blue; wet heath: blue, in deer (a) and sheep (b) across a sample of 90 consecutive hours within season. The group of lines with higher values within species is the winter season.

Thermal stress varied consistently between vegetation types across seasons (vegetation x season: *F*_4_, _4915_ = 0.625, *P* = 0.645) and between species across vegetation types and seasons (species x vegetation x season: *F*_4_, _4915_ = 0.425, *P* = 0.791) (Fig 2). Thermal stress for deer was always higher than for sheep across any vegetation type and season (species: *F*_4_, _4915_ = 18872, *P* < 0.0001), although the magnitude of the differences between species decreased in summer (species x season: *F*_4_, _4915_ = 2382, *P* < 0.0001). In general, vegetation types ranked for thermal stress as follows, bracken < grass < dry heath < rushes < wet heath (Fig 2), with significant differences between most vegetation types in deer but only between bracken and rushes and bracken and wet heath in sheep (species x vegetation: *F*_4_, _4915_ = 3.5, *P* = 0.007). There was a general consistency in the ranking of thermal stress across vegetation types in both species. Deer showed higher intra-specific variability of thermal stress than sheep (Fig 2).

### Daily distance travelled

**Fig 3:**
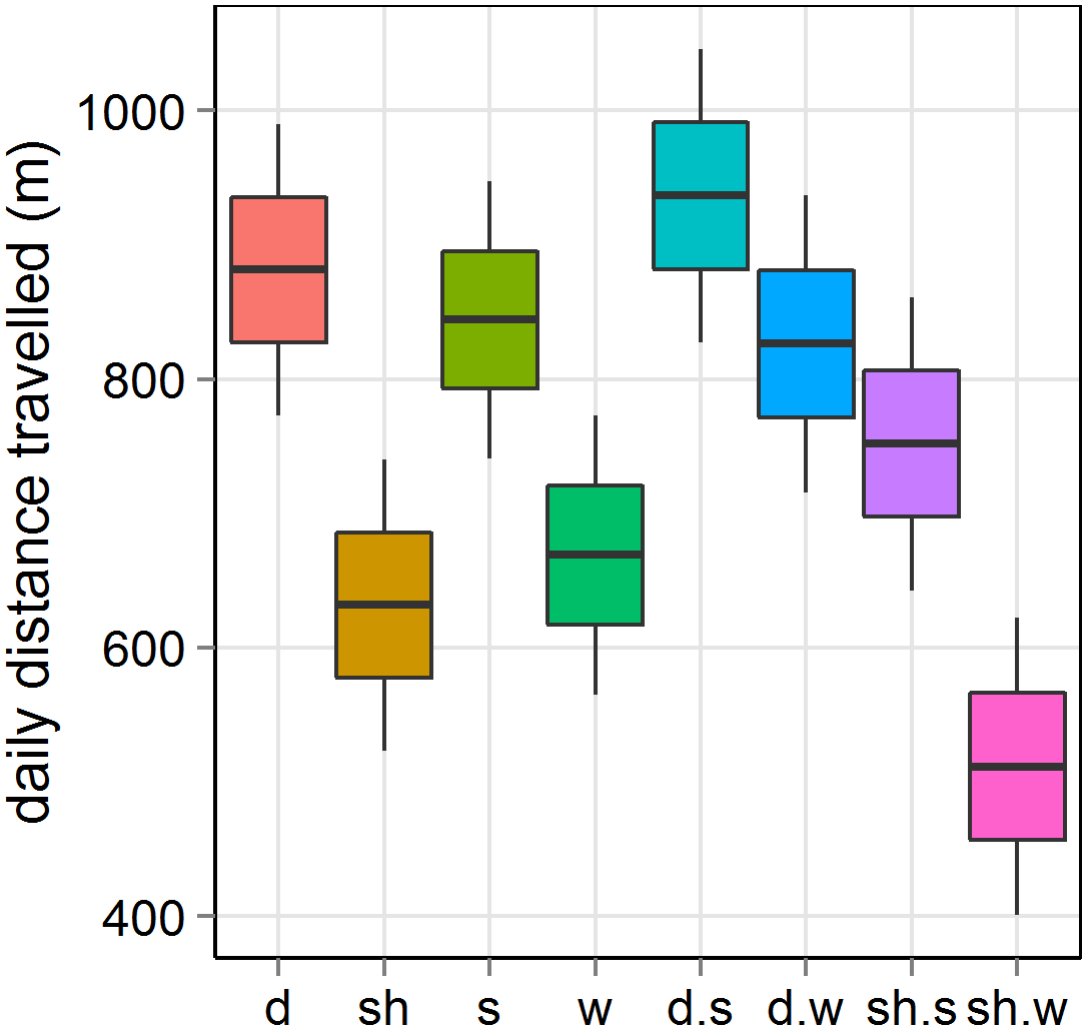
Predicted daily distance travelled by sheep (sh) and red deer (d) in summer (s) and winter (w) of the mixed linear model in Table 1. Boxes are colour coded following labels of x-axis.

**Fig 4:**
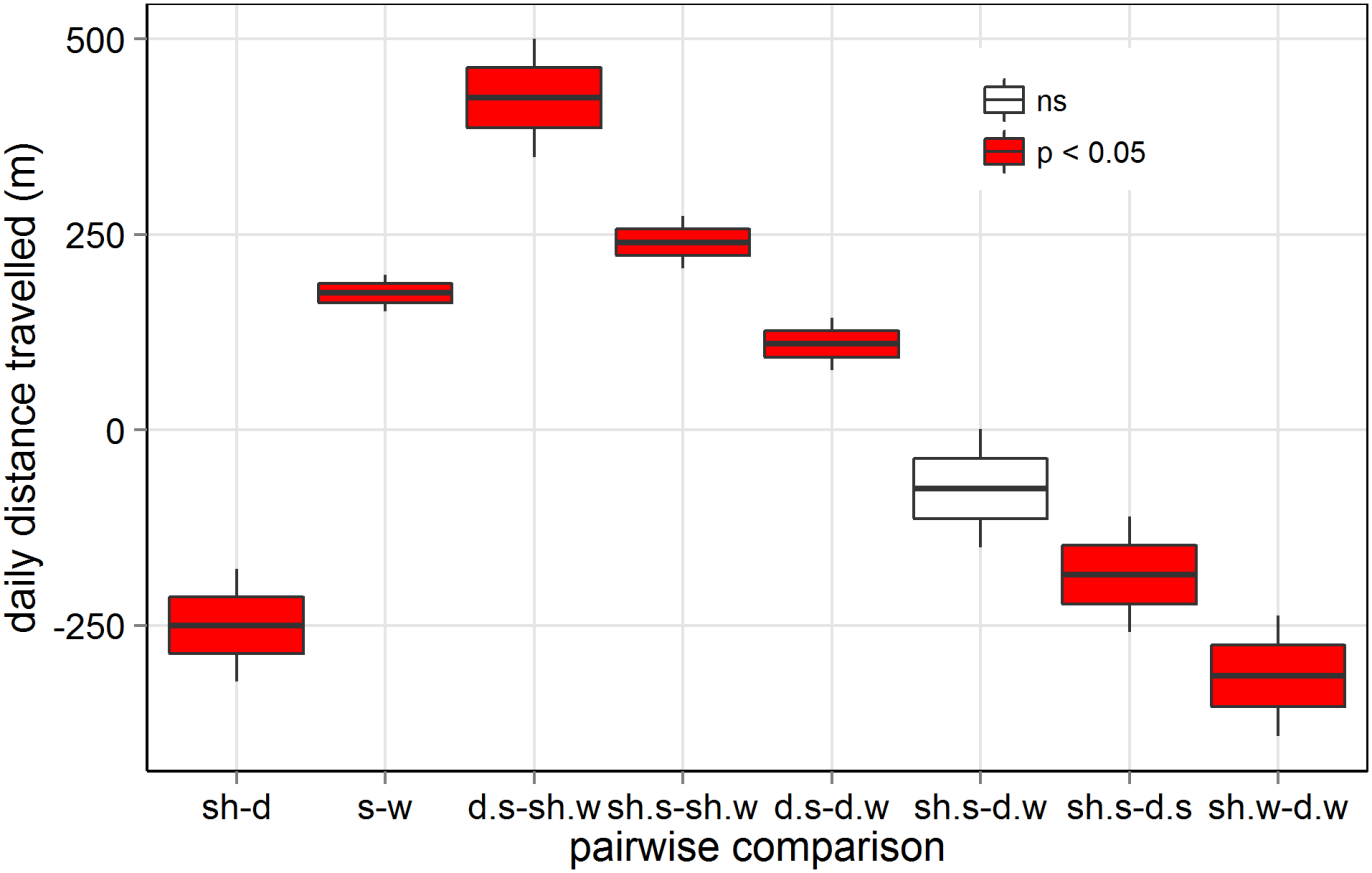
Pairwise comparisons between the predicted daily distances travelled by sheep and red deer in summer and winter (Fig 3 and Table 1). Significant levels after Bonferroni correction. Acronyms as in Fig 3.

There was a significant interaction between species and season (p < 0.0001, Table 1), indicating that seasonal differences in predicted daily distance travelled were higher in sheep (46%) than in deer (13%) (sheep summer vs winter: 752–511 m/day; deer summer vs winter: 936–826 m/day, Fig 3). Certain animals were more prone to travel longer distances than others ($\chi_{1}^{2}$ = 2513, p < 0.0001, Table 1). In average, deer travelled 40% more distance than sheep (881 vs. 632 m/day, Fig 3), and both species travelled 26% more in summer than in winter (844 vs. 669 m/day, Fig 3). Pairwise comparisons between distance travelled between species and seasons differed significantly, except sheep in summer vs. deer in winter (Figs 3 and 4).

**Table 1:** Coefficients of the linear mixed-effects model on daily distance travelled (m) by sheep and deer in summer (May to November) and winter (December and January to April) and variance accounted for random and fixed effects. Reference levels are sheep and summer.

**Random effects**				
	variance	std dev	χ^2^ (df = 1)	p
animal (intercept)	107447	327.8	2513.00	<0.0001
Residual	934015	966.4		
**Fixed effects**				
	estimate	std error	t-value	p
Intercept	751.74	54.48	13.7980	<0.0001
species (deer)	184.74	37.6	4.9130	<0.0001
season (winter)	-240.1	17.02	-14.108	<0.0001
species x season	129.83	24.07	5.394	<0.0001

### Intra- and inter-specific interactions in animal movement

**Fig 5:**
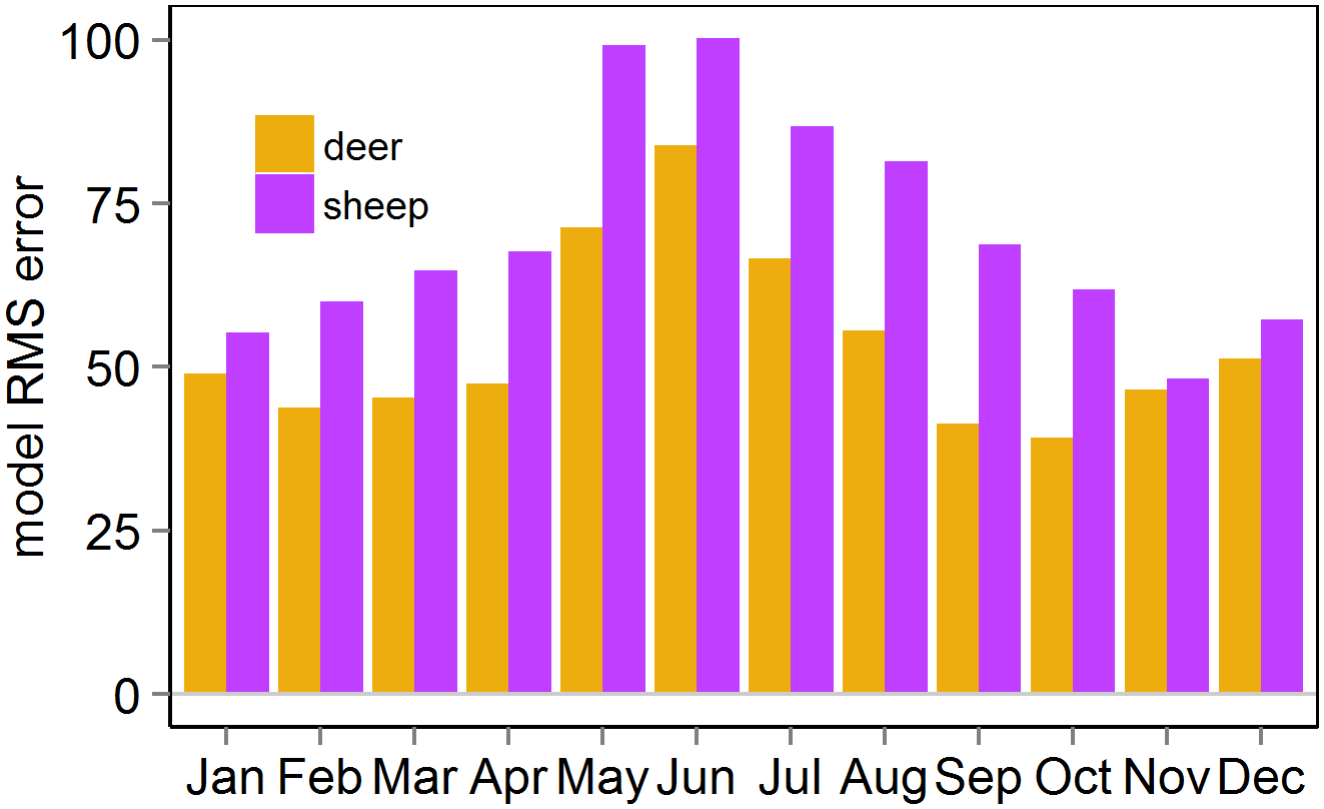
Monthly predictability (root mean square error RMS) of the movement in sheep and red deer. The plot reports systematic bias to those errors, which reflects an average global behaviour inferred from the data.

Rooth mean square error (RMS) was systematically higher in sheep than in deer (Fig 5 and Table 2), which indicates that the model is less efficient to capture the sheep movement, and the predictability of the movement followed a seasonal pattern in both species, higher in winter than in summer. This is not just dependent on the distance animals move (i.e. moving further would lead to larger predicted distances and hence larger errors), as (i) in winter movement predictability was higher than in summer, coinciding with shorter distances travelled in winter than in summer, but deer movement was better predicted than sheep movement, despite deer travelled longer distances than sheep; (ii) the values depicted in Fig 5 are maximum likelihood estimates that indicate that the seasonality variation is systematic, and the intra-specific variation is consistent across the year.

**Table 2:** Parameters of the models for sheep and red deer, respectively. Root mean square error (RMS). Model size is proportional to the number of parameters in the model fitted to the data. Minimum Description Length (MDL), is the metric used to select from among competing models (i.e. it is the length of the compression of the data one achieves by using the model). Number of observations (n), number of times one records a useable measurement of an animal of a given type and sufficient number of neighbours in the stated environment. The basic functions of the models were Gaussians, wavelets (of a particular type) or tophat (sharpened/flattened Gaussians).

model		model size	MDL	RMS	n
		328–1313	154226–270239	65.5–51.7	26972–47888
month	Jan	261–541	68874–100704	55.2–48.6	12214–17954
	Feb	217–652	59317–105537	59.8–43.8	10382–19022
	Mar	117–574	41719–93704	64.7–45.0	7268–16796
	Apr	130–899	57074–141886	72.2–47.5	9800–25102
	May	210–640	128541–140409	99.21–71.2	21032–23578
	Jun	195–483	138679–140113	100.4–84.0	22694–23140
	Jul	236–445	96458–105266	86.6–66.4	16016–17970
	Aug	234–687	96176–129705	81.4–55.5	16148–22602
	Sep	152–479	55487–74192	68.4–41.0	9576–13530
	Oct	116–525	35475–65920	61.9–39.1	6198–11894
	Nov	408–536	105962–93557	48.5–46.5	19252–16772
	Dec	216–483	97986–99657	57.4–51.3	17538–17714
vegetation cover	dry heath	645–2408	463726–515848	83.0–59.0	78372–89458
	bracken	141–298	112400–134801	93.4–84.1	18644–22546
	rushes	63–198	34519–65467	91.4–81.9	5716–10908
	grass	86–238	61711–58519	114.1–66.9	9890–10000
	wet heath	2–706	572–163974	130.4–63.1	88–28196
thermal stress	summer TS	508–737	60900–58298	53.2–32.6	10352–10304
	Winter TS	859–1055	83852–90322	28.72–23.2	15748–17340
	summer	484–715	61320–61135	55.1–34.9	10408–10784
	winter	828–1075	84114–93262	29.3–23.7	15780–17852

**Fig 6:**
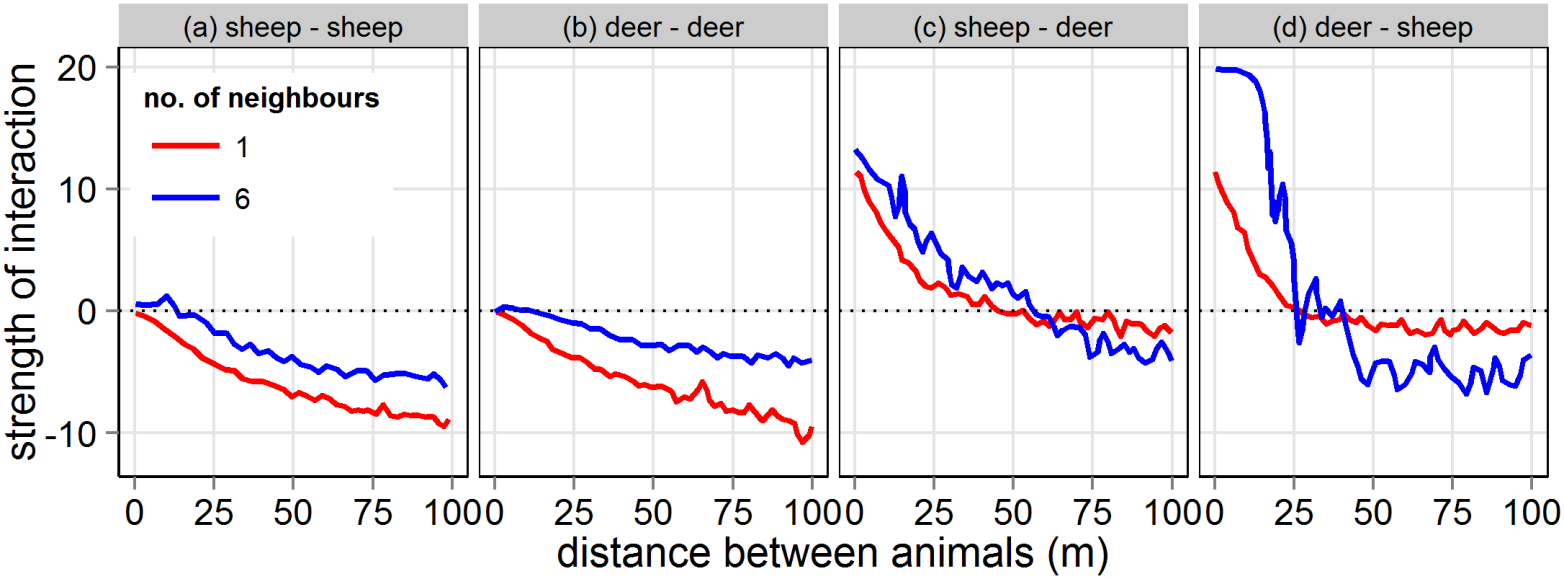
Computational estimation of the direction and strength of interaction (negative values is attraction, positive values is repulsion) of an animal as a function of the distance to its target neighbours. For simplicity we only include the closest (red line) and the six closest (blue line) neighbours.

The model estimated the mathematical structure of the dynamical behaviour of the animals’ movement as follows: when separated by a greater distance animals tended to be attracted to each other, but attraction weakened as the separation distance got shorter (Fig 6). The strength of this behaviour changed depending on whether the interactions are intra- or inter-specific. At greater separation distances the inter-specific attraction is weaker than the intra-specific attraction, but at shorter separation distances (<50 m) the repulsion between species was stronger than the repulsion between animals of the same species (Fig 6). This pattern was consistent in the range from one target animal up to six target animals (i.e. the maximum number of target animals included in the model, Fig 6). Repulsion at short separation distances and attraction at greater separation distances were both reinforced when the number of neighbours of the other species increased, and deer were more influenced than sheep by this effect (Fig 6c and 6d).

The asymmetrical property of the model allowed us to assess which species attracted or repelled the other. For example, by comparing Fig 6c against Fig 6d (red line: strength of interaction between the closest animal) there was no strong evidence that one species was more attracted or repelled by the presence of the other species.

**Fig 7:**
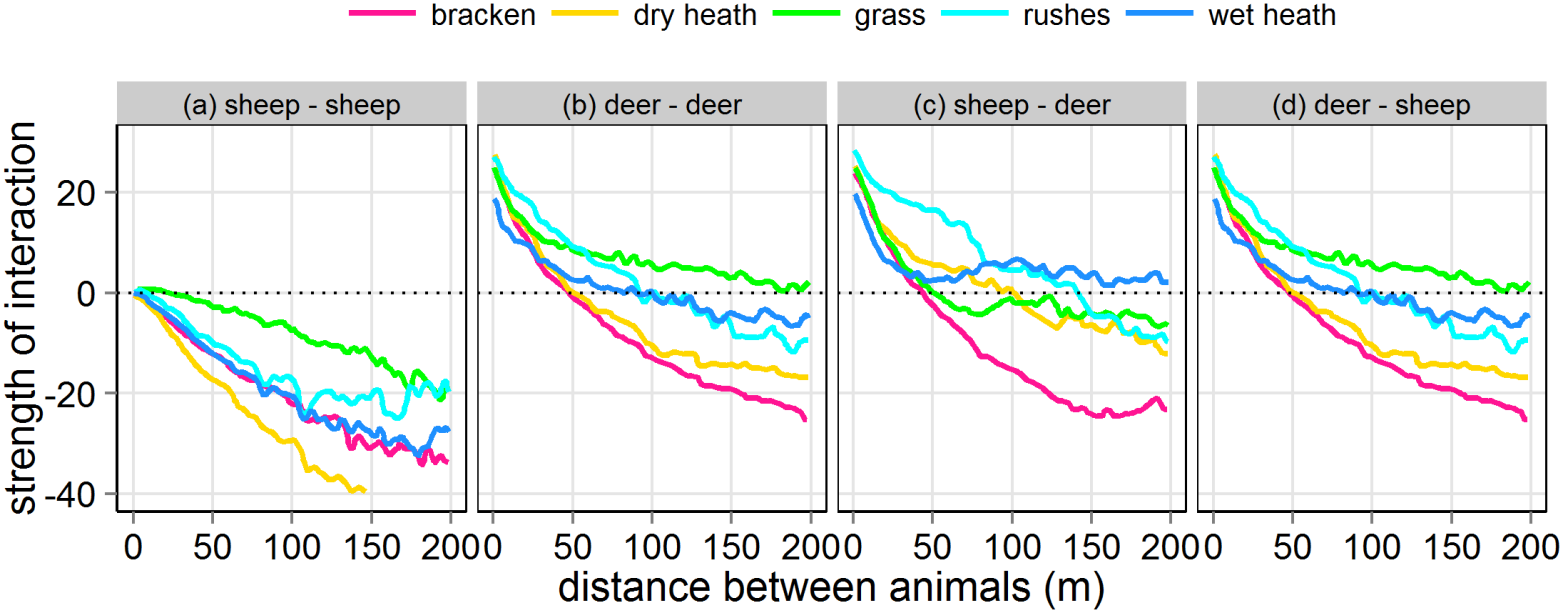
As Fig 6 but including the effect of vegetation type. For simplicity we included only interactions with the nearest neighbour.

The structure of the dynamic behaviour of the animals’ movement, described above, was consistent across all vegetation types (Table 2). The intra-specific attraction was weaker in grass than in dry heath, with intermediate values in rushes, wet heath and bracken (Fig 7a and 7b). Vegetation type had little effect in inter-specific interactions, with the exception of bracken, in which species attracted each other at distances of >50m (Fig 7d). These patterns can also be interpreted in terms of tendency to move. For example, sheep moved less once they are on grass in comparison with dry heath.

**Fig 8:**
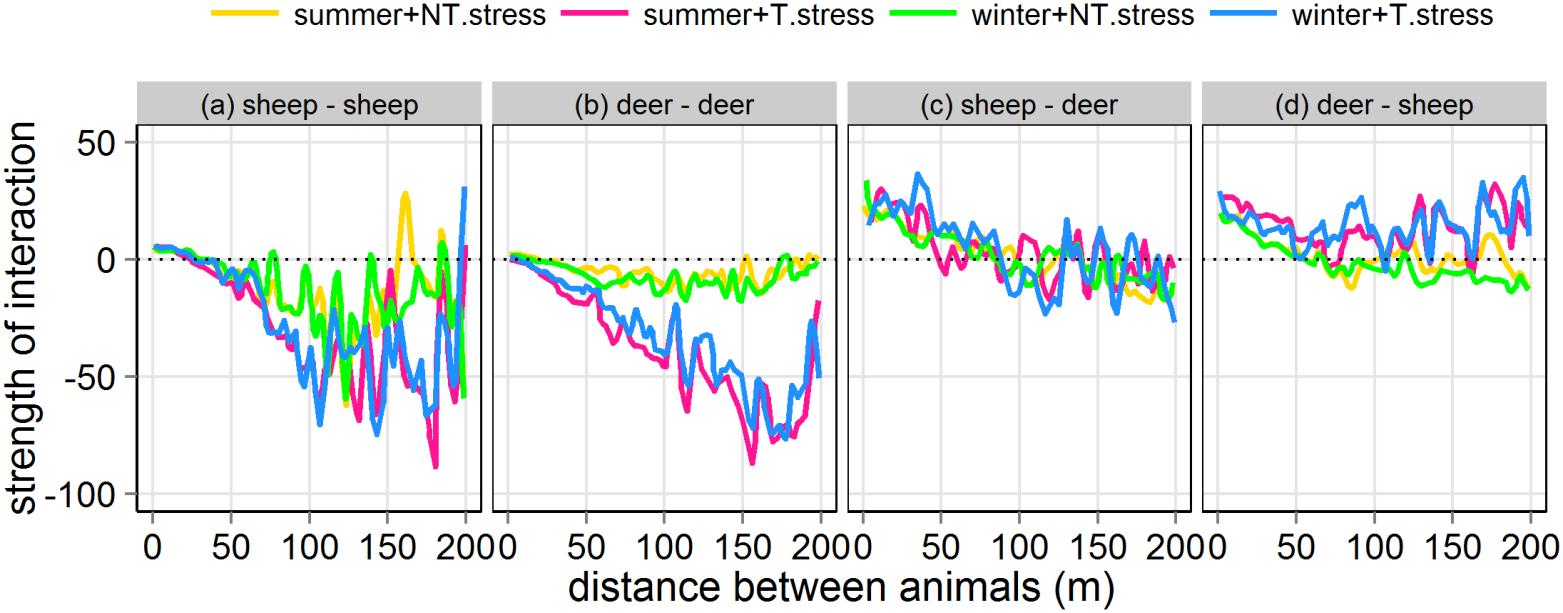
As Fig 6 but including and not including the effect of thermal stress in summer and winter.

The structure of the dynamic behaviour was hardly affected by the thermal stress that the animals experienced across the experimental plot, independent of season (Fig 8 and Table 2). Only intra-specific interactions for deer showed differences in structure when thermal stress was included in the model; attraction between deer at greater separation distances was stronger when the model accounted for thermal stress (Fig 8b), probably because there were fewer places in the landscape that offered shelter from thermal stress.

## Discussion

Our results indicate that (a) thermal stress was higher in deer than in sheep, with less differences between species in summer; (b) deer travelled more distance than sheep, and both species travelled more in summer than in winter; deer travel distance showed less seasonal differences than sheep; (c) animal movement was better predicted in deer than in sheep and in winter than in summer for both species; (d) both species showed a swarming behaviour, but this was stronger in deer; at shorter separation distances swarming repulsion was stronger between species than within species, but at greater separation distances inter-specific attraction was weaker than intra-specific attraction; (e) there was a positive density-dependent effect on swarming, and stronger in the deer than in sheep; (f) there was not clear evidence which species attracted or repelled the other; (g) attraction between deer at greater separation distances was stronger when the model accounted for thermal stress, but in general the dynamic movement behaviour was hardly affected by the thermal stress experienced by the animals; (h) vegetation type affected intra-specific interactions but had little effect on inter-specific interactions.

These results demonstrate the importance of the use of models taking into account the local and time specific changing conditions to assess the drivers of animal movements, both within and between species. Stewart *et al* [48] assessed niche overlap in *Cervus canadensis*, *Odocoileous hemionus* and cattle; they found that species interference was the strongest explanatory factor of habitat use in their data. Methods that use the static distribution of animals, such as factor analysis or resource selection functions, try to estimate fitness functions for various habitats, as the resource-use patterns are a consequence of the influence of selection on survival and reproduction [79]. In general, these methods attempt to predict animal distribution at large spatial scales, which makes it difficult to assess the drivers of movement of animal species that share similar resources. Another static approach to explain the coexistence of grazing herbivores at finer spatial scale is the use of differences in body mass and bite size in relation to sward height, smaller species benefit from grazing in areas already used by larger grazers where sward height is too short for large grazers to maintain their levels of intake [80]. These models use the distribution of a mosaic of food resources to explain the presence of different species that differ in body mass, but they are not designed to use the knowledge of the dynamics of the current state to predict their movement.

The structure of the motion unveiled by our models suggests trade-offs between competition (probably for food and space) and social interactions. This was supported by the findings that at greater distances of separation animals tended to attract each other. As the distance of separation got shorter attraction weakened and repulsion got stronger, especially between species. This is in agreement with our prediction of swarming behaviour (H1), which seems to be consistent across other social vertebrate taxa, as fish, birds and mammalian species [53–55,76]. It is also possible that the fact that species tend to maintain their social spatial structure contributes to the spatial separation from other, taxonomic close, species, as it has been suggested to explain sexual segregation in ungulates [51,81].

We hypothesised that swarming behaviour should vary between different vegetation types, as a consequence of species preference for certain type of vegetation (H2) hampering inter-species interactions. However, this was not corroborated across all vegetation types in our plot, and in the cases that it was corroborated the effect was not strong. Although vegetation represents a food resource which is generally of low quality and difficult to defend due to its wide spatial distribution and temporal variability [82–84], antagonistic behaviour for access to patches of food has been observed in ungulates in the wild [85–90]. In our study attraction between peers of the same species was only stronger in dry-heath than in grass, this could be due to the patchy distribution of grass in comparison with the larger and homogenous areas occupied by dry heath in our study plot. It would also be expected that, in these small preferred patches of vegetation, intra-specific attraction would be density dependent, but this was not the case. The animal densities used in our plot are similar to high densities of wild deer and sheep in extensive systems in the Scottish Highlands [63,91]. It is difficult to know if these densities are saturating the vegetation types of our plot, hampering the effects of density and vegetation use on animal movement. However, these densities were certainly not high enough to hamper the effect of density on swarming behaviour, as our results indicate.

Consistent with our results, Kattas et al [41] found that the movement of deer and sheep was coupled, especially when sheep formed groups. They suggested that sheep tend to follow deer, but this was not corroborated by our results. In our study the forces of attraction and repulsion between species are fairly symmetric between the nearest neighbours, which indicates that the movement behaviour of both species responds in a similar way to the other species presence, with the only difference being that deer were more responsive to the presence of sheep when sheep formed groups (i.e. deer were more repelled by groups of sheep at shorter distances, and more attracted to groups of sheep at greater distances, than sheep were to groups of deer).

Some studies have reported changes in activity related to thermal stress, such that animals optimize their energy budget in different weather conditions by changing their spatial and temporal behaviour patterns [24,25,92]. In our study, we predicted that red deer would suffer approximately 1.6 times higher thermal stress than sheep, as indicated by the electrical consumption of our heated mechanical models and from the difference in insulation coat thickness between species [56]. However, the inclusion of thermal stress in our models only affected the intra-specific interactions in deer, but had no effect on inter-specific interactions. This suggests that species differences in exposure to thermal stress were not enough to override the structure of the motion that was determined by species interactions. It is also possible that our plot did not offer enough variation in thermal stress, as to allow each species to display significant changes in motion structure.

A plausible explanation for the lack of thermal stress effect in our study may have been that it is too variable across time and space, which made it difficult for sheep and deer to track and utilise the most benign thermal environment. In addition, animals can use micro-topographic features (e.g. vegetation, boulders, ground depressions) to shelter from rapid changes in thermal stress [27], rather than search for a less stressful thermal environment. This is particularly the case in habitats where there is a match between the spatial distribution of high quality food and harsh thermal stress, i.e. animals would seek for micro-topographic features to shelter from harsh weather to stay close to food resources of high value [93].

We detected species and seasonal effects on the deterministic movement of the animals. The models were better at capturing the movement of deer than the movement of sheep, despite the fact that deer travelled longer distances than sheep. This is unlikely to be because sheep suffer less thermal stress than do deer, as the mechanical models indicate. Nor do sheep appear to be better at finding shelter provided by topography and vegetation than deer, as we did not detect any species x season interactions in movement behaviour, although the interaction was significant in distance travelled (i.e. sheep should have a less deterministic movement behaviour in winter, however, both species had a similar deterministic movement in summer). Among the plausible explanations for our findings are that sheep might have a less deterministic pattern of movement as a consequence of the larger number of sheep in the plot, making them less proportion of animals GPS tracked, in comparison with the number of deer, this increases the number of interactions between sheep making their decision process more complicated and so less predictable than in deer. A less deterministic behaviour is also possible by individualist behaviour (i.e. individuals obeying separate and distinct preferences from its peers), then the larger number of sheep would also have the effect of making them appear less deterministic. It is also possible that, as sheep have a greater preference for grazing on small patches of grass in comparison with deer [94], and small grass patches are spread across a heather matrix, their movement is less deterministic than that of deer; deer might concentrate their activity on a few large patches of grass, or use a mixture of grass and heather patches across the plot [95]. This latter explanation cannot be tested using our data set, because the grain of the grass mosaic within heather is too fine for the 1 hour interval time of our GPS fixes.

The deterministic nature of the movements of both species had a seasonal pattern, more deterministic in winter than in summer. This could be due to a reduction in the area of benign thermal stress habitat in winter (i.e. less use of the areas exposed to harsh weather), which coincides with the season when both species travelled less, which makes the movements more predictable than in summer, when primary production peaks and allows both species to utilise the area extensively.

## Supporting Information

S1 Data Hourly animal movement (N: Northings; E: Eastings), species (deer, sheep), animal ID (ear_tag) and habitat (Veg_Summ). (ZIP)

S1 Fig Plots of the components of the smooth functions of the GAM model on the scale of the linear predictor (air temperature, wind speed, income solar radiation, rainfall and humidity) and fitted values for the sheep heated mechanical model. Prediction (black line), standard errors of the prediction (shade) and partial residuals (dots). The predictions of the model (fitted values) were calculated for the intervals 0.59–1.14 W/h/kg^0.75^.
(TIF)

S2 Fig Plots of the components of the smooth functions of the GAM model on the scale of the linear predictor (air temperature, wind speed, income solar radiation, rainfall and humidity) and fitted values for the deer heated mechanical models. Prediction (black line), standard errors of the prediction (shade) and partial residuals (dots). The predictions of the model (fitted values) were calculated for the intervals 0.4–2.1 W/h/kg^0.75^.
(TIF)

S1 Table Description of the experimental plot and area of the predominant vegetation types and statistics of their mosaic structure (area in m2). Mean, minimum (min), maximum (max), standard deviation (sd) and number (n) of the patches of the different types of vegetation.
(DOCX)

S2 Table General additive regression model to predict the electrical consumption of heated mechanical models of sheep and deer. Values are given in pairs, sheep and deer, separated by a hyphen. edf: estimated degrees of freedom; rdf: estimated residual degrees of freedom. N: number of hourly records.
(DOCX)

S1 Text Supporting information on microclimate and heated mechanical models. (DOCX)

S2 Text Details on the computational modelling and approach and algorithms used in the paper. (PDF)
