# Supplementary figures and images for: State-Space Modelling of the Drivers of Movement Behaviour in Sympatric Species
Source: PLoS One. 2015 Nov 18;10(11):e0142707. doi: 10.1371/journal.pone.0142707 (PMC4651358; doi:10.1371/journal.pone.0142707)

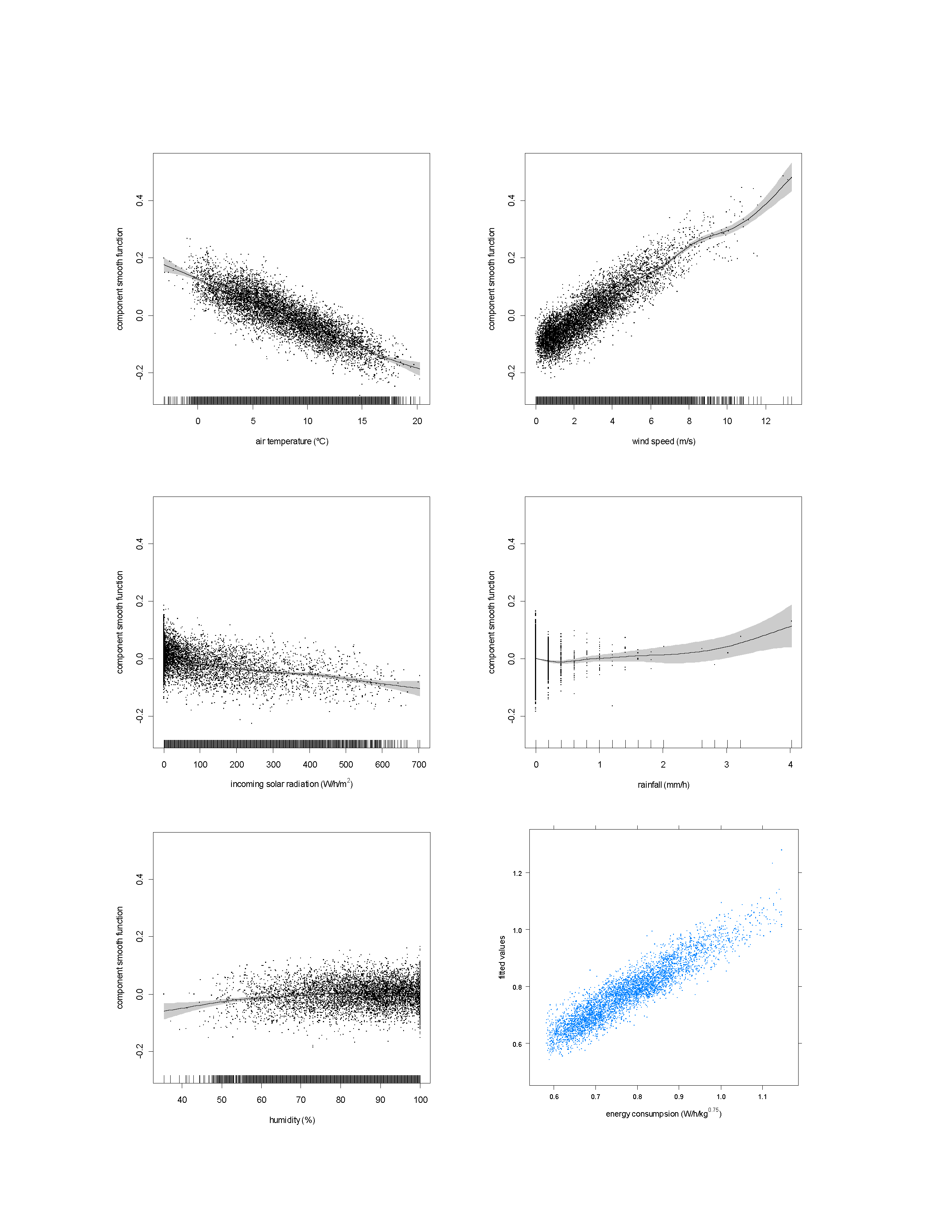

Supplement: S1 Fig — Prediction (black line), standard errors of the prediction (shade) and partial residuals (dots). The predictions of the model (fitted values) were calculated for the intervals 0.59–1.14 W/h/kg0.75. (TIF) [file pone.0142707.s002.tif]

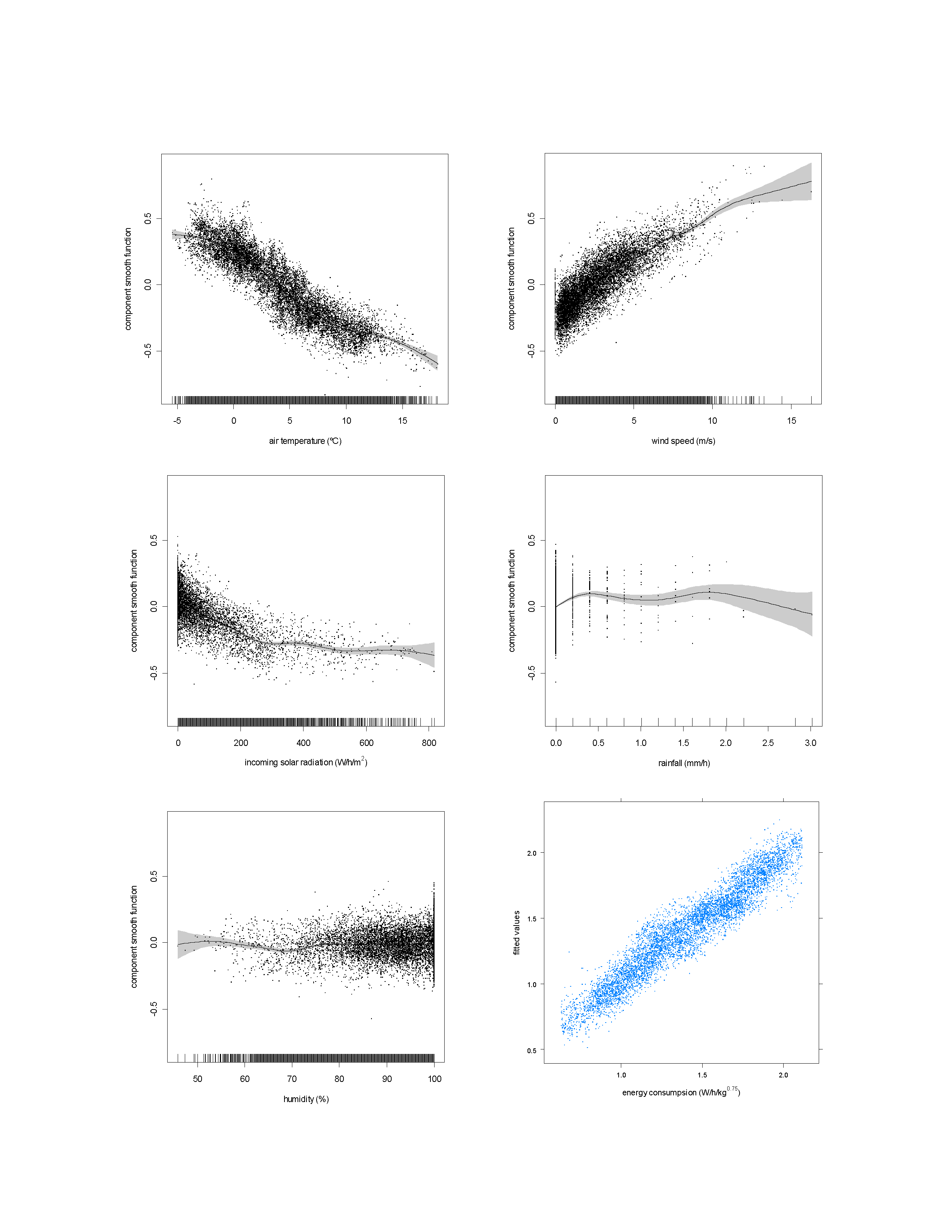

Supplement: S2 Fig — Prediction (black line), standard errors of the prediction (shade) and partial residuals (dots). The predictions of the model (fitted values) were calculated for the intervals 0.4–2.1 W/h/kg0.75. (TIF) [file pone.0142707.s003.tif]
