# Supplementary material for: State-Space Modelling of the Drivers of Movement Behaviour in Sympatric Species
Source: PLoS One. 2015 Nov 18;10(11):e0142707. doi: 10.1371/journal.pone.0142707 (PMC4651358; doi:10.1371/journal.pone.0142707)
Supplement: S1 Table — Mean, minimum (min), maximum (max), standard deviation (sd) and number (n) of the patches of the different types of vegetation. (DOCX) [file pone.0142707.s004.docx]

| vegetation  type | description | area | mean | range | sd | n |
| --- | --- | --- | --- | --- | --- | --- |
| Bracken | Area dominated by *Pteridium aquilinum,* with patches of *Vaccinium myrtillus, Deschampsia flexuosa, Anthoxanthum odoratum.* | 79649  (7.7%) | 13275 | 205-53828 | 21041 | 6 |
| Dry heath | Principally common heather (*Calluna vulgaris*), bell heather (*Erica cinerea*), blackberry (*Vaccinium myrtillus*), and abundant graminoids like *D. flexuosa*, *A. odoratum*, *Agrostis capillaris* and *Juncus squarrosus.* | 498766  (48.1%) | 27709 | 60-243897 | 72724 | 18 |
| Grass | Predominantly areas of acid grassland, containing *Festuca ovina, Agrostis capillaris, Galium saxatile.* | 34859  (3.4%) | 4357 | 27-14620 | 5776 | 8 |
| Rock | Areas of exposed rock or loose stones. | 12028  (1.2%) | 668 | 23-4089 | 987 | 18 |
| Rushes | Areas with an abundance of either *Juncus effusus* or *J. acutiflorus* sometimes both, in a ground of mesophytic herbs and moss. | 26856  (2.6%) | 1790 | 45-11216 | 3200 | 15 |
| Wet heath | Ground dominated by mixtures of *Eriophorum vaginatum* and *Trichophorum cespitosum*, and ericoid sub-shrubs (mainly *C. vulgaris*, *Erica tetralix* and *Empetrum nigrum*), with *Sphagnum* and *Polytricum* mosses present in wetter areas. | 384050  (37.1%) | 192025 | 460-383591 | 270914 | 2 |
| Total |  | 1036207 | 15466 | 23-383591 | 60141 | 67 |
