# Supplementary material for: State-Space Modelling of the Drivers of Movement Behaviour in Sympatric Species
Source: PLoS One. 2015 Nov 18;10(11):e0142707. doi: 10.1371/journal.pone.0142707 (PMC4651358; doi:10.1371/journal.pone.0142707)
Supplement: S2 Table — Values are given in pairs, sheep and deer, separated by a hyphen. edf: estimated degrees of freedom; rdf: estimated residual degrees of freedom. N: number of hourly records. (DOCX) [file pone.0142707.s005.docx]

| Parametric coefficients  *N* = (10684 - 11650) | | | | |
| --- | --- | --- | --- | --- |
|  | estimate | SD error | *t* | *P* |
| intercept | 0.7-1.4 | 0.0004-0.001 | 1775-1395 | <0.001-<0.001 |
| Approximate significance of smooth terms | | | | |
|  | edf | rdf | *F* | *P* |
| body temperature | 4.2-8.7 | 5.3-9.0 | 13-37 | <0.001-<0.001 |
| air temperature | 7.9-8.7 | 8.7-9.0 | 2158-4517 | <0.001-<0.001 |
| wind speed | 8.0-9.0 | 8.7-9.0 | 2184- 2454 | <0.001-<0.001 |
| solar radiation | 5.6-8.0 | 6.8-8.7 | 463-727 | <0.001-<0.001 |
| humidity | 6.8-7.7 | 7.8-8.5 | 16-38 | <0.001-<0.001 |
| rainfall | 4.4-5.0 | 5.2-6.0 | 7-29 | <0.001-<0.001 |
