# Supplementary material for: State-Space Modelling of the Drivers of Movement Behaviour in Sympatric Species
Source: PLoS One. 2015 Nov 18;10(11):e0142707. doi: 10.1371/journal.pone.0142707 (PMC4651358; doi:10.1371/journal.pone.0142707)
Supplement: S1 Text — (DOCX) [file pone.0142707.s006.docx]

# Microclimate data

We recorded air temperature and wind speed by deploying 17 portable meteorological stations (Davis Weather Wizard III; www.davisnet.com) within the plot and in the surrounding area (Fig. 1 in the printed paper version). The location of the stations took into account the topographic variability of the plot in terms of altitude, slope, and aspect. The console and logger of the meteorological stations were kept in a waterproofed case, cables were plastic-pipe protected from rodents and the temperature probe and anemometer were fixed to a pole at 85 cm above ground (average flank height of the animals). The meteorological stations measured parameters every 4s and recorded values averaged over 1 hour time periods. Data from the loggers were manually downloaded once a month, at which time meteorological stations were also checked and batteries replaced.

In addition, we deployed a meteorological station nearby at Birnie Hill (Fig. 1 in the printed paper version) fitted with a HMP45c relative humidity probe (Vaisala), ARG 100 tipping bucket rain gauge (Environmental Measurements Ltd) and a CM3 thermopile pyranometer (Kipp and Zonen) that recorded incident (direct and diffuse) solar radiation. Hourly records of relative humidity, rainfall and incident solar radiation were stored in a CR10X data logger (Campbell Scientific) and sent via mobile phone technology to The James Hutton Institute in Aberdeen every two hours. We assumed that relative humidity and rainfall did not vary much across the plot, so one spatial point was representative of the whole experimental plot for these parameters.

Hourly incoming solar radiation was estimated by combining theoretical data calculated using Area Solar Radiation tool (ASR) in ArcMap 9.3 and empirical data recorded at this meteorological station. ASR calculates potential radiation (W/m^2^) in clear sky conditions at the surface at the local scale using digital elevation models for input and algorithms that account for the influences of the viewshed, aspect, elevation and effects of shadows cast by surrounding topography.

We calculated incident direct and diffuse radiation for each 10m×10m cell of a 10km x 10km area centred on the study site. A 10km x 10km area ensured that all shadows cast by surrounding topography that affected the solar radiation received by our plot were included in the ASR computations. Topographic data were taken from a digital elevation model (Ordinance Survey, LandForm Profile) with 10 m × 10 m horizontal resolution. TThe incoming diffuse sky radiation was assumed to be the same from all directions. Transmittance of the atmosphere and the proportion of global normal radiation flux that is diffuse were set to 0.5 and 0.3, respectively, in order to represent a generally clear sky. Actual hourly changes in atmospheric conditions (cloudiness, haze) were accounted for by rescaling the values of the ASR model by a correction factor. The correction factor at time *t* was calculated as the relationship between the ASR insolation value at the pyranometer point and the actual reading of the pyranometer. We assumed that the radiation by surface unit across the plot was the same as the radiation recorded at the nearby pyrometer location.

## Thermal stress and heated mechanical models

We built two heated mechanical models using aluminum hollow cylinders (aluminum sheet 1mm thickness) of the same volume of our average deer and sheep (deer: 97 cm x 40 cm; sheep: 80 cm x 32 cm). Tanned winter hides of hinds and ewes of similar age to our animals were glued and sewn to the cylinders. Inside the cylinders three heater mats (200 mm x 150 mm, 200 W each) were fitted around a metal mesh cylinder of 10 cm diameter along the longitudinal axis of the model and a small internal fan facilitated heat circulation. A temperature sensor was suspended between the heater mat and the aluminum wall of the model and a thermostat fitted outside the model to allow control of the internal temperature. The thermostat was set at 39.7 ⁰C, which was the average rectal temperature of our animals (measured across the year on different dates and time of the day). The models were mounted on a rotary ball-bearing pole and fitted with a vane, so that the major axis of the model was always perpendicular to the wind direction. Although the typical animal behaviour is facing cold winds to minimise heat loss, we opted for assuming perpendicular position to the wind direction to be conservative in our estimates of thermal stress. The joins between pole, cables and the model, and the hide seams were properly insulated to minimise heat loss. A handheld infrared thermometer was used to ensure that heat loss through the seams was similar to heat loss in the adjacent regions

The models were deployed in a small fenced enclosure near the experimental plot (Fig. 1 in the printed paper version) together with a meteorological station similar to the one in Birnie Hill, where mains electricity was available, so the models could be run continuously year round. The data logger of the meteorological station also stored information on the electrical power consumption of the models, and all information was sent to The James Hutton Institute in Aberdeen via a mobile phone link every two hours.

## Thermal stress index

We found that the core temperature of the mechanical models fluctuated around the 39.7⁰C setting (interquartile: deer = 0.6⁰C, sheep = 1.3⁰C). Therefore, we adjusted heater power to a fixed core temperature of 39.7⁰C in both animal models using a GAM with the predictors (meteorology variables) as smooth terms with automatic selection of the effective degrees of freedom. Consequently, our thermal stress index was defined as the electrical consumption of the heated mechanical model (sheep or deer) to maintain 39.7⁰C in the core temperature of the model under certain thermal environment. The units of the index were W/BM^0.75^ (where BM was the average body mass in kg of our animals by species).

Note that the heater power required by the mechanical models was not calibrated against measured values of metabolic heat production of sheep and deer from calorimeter trials [1]. Consequently, our predicted heater power for the models is an index of comparative thermal stress, not an estimate of actual metabolic demands [2].

## Microclimate parameters

Because we had only 17 point measurement of meteorological data (or a minimum of 15 in case of malfunction of some of them), but needed a value for the thermal stress index at each 50 m × 50 m cell of the study area, the information from the 17 meteorological stations was interpolated to create 1 and 24 hour spatial surfaces of each variable. We used multivariate and geostatistical techniques, following the approach in Poggio *et al* [3] to take into account the effect of the topography of the plot (elevation, slope and aspect) in the interpolation of the meteorological data.  This approach extends regression kriging [4] and decomposes the data into a non-linear trend and a local component, thus addressing the problem of non-stationarity of the spatial mean. The first is modelled using a Generalized Additive Model (GAM), the second using kriging on the GAM residuals. The two components are modelled separately and then added together.

The core of the analysis was to apply a GAM to each of our meteorological variables using elevation, slope and a linear transformation of aspect as the explanatory variables. To circumvent the problem that we had three explanatory variables and only a maximum of 17 spatial points for the regression model (i.e. the number of meteorological stations), we applied a principal components analysis (PCA) to the three explanatory variables to summarise their information in a single variable. We used the first principal component as a smooth term in a GAM (a thin plate spline regression), with automatic selection of the effective degrees of freedom for the smooths [5]. To provide the spatial component we also used a bidimensional smoother of the x and y coordinates of the meteorological stations as an independent variable [5].  We used the fitted GAM model, together with the covariates values at unsampled locations, to predict (interpolate) the general trend of the meterorological variable of interest over a grid of 10 m cell across the plot.

The variogram of the GAM residuals was then modelled, and a choice between exponential or spherical model was made, based on Akaike’s information criterion. The residuals were kriged, obtaining values for the same locations as those of the aforementioned 10 m grid. Finally, the 2 grids were added together, to obtain 10 m resolution estimates of the meteorological variable for the study area. For ease in modelling the variograms, and for consistency when adding together the 2 components, we used normal-score transforms of all the variables involved and then back-transformed the results [6].

We used *geoR*, *mgcv*, *gstat*, *MASS* and *adehabitat* packages from R language and environment for statistical computing [7].

The resulting kriged spatial surfaces of each variable were then used as input data for the GAM model predicting hourly electrical consumption by the heated mechanical models to generate the final spatial surface of heater power across the study area at a resolution of 10 m × 10 m. This microclimate index will be referred to as 'thermal stress’ hereinafter.

Reference List

1. Bakken GS, Reynolds PS, Kenow KP, Korschgen CE, Boysen AF (1999) Standardization and calibration of heated mounts illustrated with day-old mallard ducklings. Physiological and Biochemical Zoology 72: 502-506.

2. Dzialowski EM (2005) Use of operative temperature and standard operative temperature models in thermal biology. Journal of Thermal Biology 30: 317-334.

3. Poggio L, Gimona A, Brown I, Castellazzi M (2010) Soil available water capacity interpolation and spatial uncertainty modelling at multiple geographical extents. Geoderma 160: 175-188.

4. Hengl T, Heuvelink GBM, Stein A (2004) A generic framework for spatial prediction of soil variables based on regression-kriging. Geoderma 120: 75-93.

5. Wood, S. (2006) Generalized Additive Models: An Introduction with R. Boca Raton.

6. Goovaerts, P. (1997) Geostatistics for natural resources evaluation. New York.

7. R Development Core Team (2009) R: A language and environment for statistical computing, version Viena, Austria: R Foundation for Statistical Computing.
