# Supplementary material for: State-Space Modelling of the Drivers of Movement Behaviour in Sympatric Species
Source: PLoS One. 2015 Nov 18;10(11):e0142707. doi: 10.1371/journal.pone.0142707 (PMC4651358; doi:10.1371/journal.pone.0142707)
Supplement: S2 Text — (PDF) [file pone.0142707.s007.pdf]

Under the hood: Modelling Interaction with Radial Basis Function Networks and Minimum Description Length

F.J. Pérez-Barberia Michael Small

October 19, 2015

Abstract

This supplementary information for our paper *State-space modelling of the drivers of animal movement* provides additional detail of the computational modelling approach we employ. While this information is available in the cited references, we include a complete description here so that our results may be more self contained. In this document we first introduce the radial basis algorithm previously employed to model deterministic scalar time series data and then describe how this can be adapted to provide a deterministic state space model of animal movement.

1 Introduction — problem background

There are many potential approaches which one can apply to model deterministic causal relationships among observed variables. The approach we adopt here is merely that which we are most comfortable with and which we are most capable of using appropriately. Let $\{x_t\}_t$ be a sequence of scalar time series observations — that is, x_t is a single real number which is the experimentally measured output state of some, presumably, deterministic system. We will deal with both observational and dynamical noise in what follows, but the behaviour of interest to us is the deterministic interaction.

The deterministic interaction is assumed to be due to a higher (than 1) dimensional system and the scalar state x_t is merely our "read-out" of that system. To obtain a proxy of that underlying higher dimensional system — the underlying *state-space* — we appeal to Takens' Embedding Theorem [6] a linchpin of nonlinear time series analysis since the 1980s [5]. Under moderately mild assumptions (which are taken to hold as a matter of expedience) we can construct vector points v_t from delay versions of the scalar:

$$v_t = (x_t, x_{t-1}, x_{t-2}, \ldots, x_{t-d+1}) \tag{1}$$

where the embedding dimension d needs only be sufficiently large to capture

the deterministic dynamics[1]. By appealling to Takens' theorem we now have a sequence of vectors v_t such that the transition between them $v_t \to v_{t+1}$ captures the underlying deterministic dynamics. Observational noise enters the picture here and can (essentially) be thought of as replacing these observations with probability distributions, nonetheless the observed values are the maximum likelihood estimate of the underlying state[2].

A fundamental question in nonlinear time series analysis and experimental dynamical systems theory is now how can we recover the function $F : \mathbb{R}^d \to \mathbb{R}^d$ that captures the underlying dynamics of our system? That is, we require that $F(v_t) \approx v_{t+1}$. Note that since $v_{t+1} = (x_{t+1}, x_t, x_{t-1}, \ldots, x_{t-d+2})$ all of the information to obtain v_{t+1} is contained in v_t 1 — except x_{t+1} and hence, what we actually require is a function $f : \mathbb{R}^d \to \mathbb{R}$ such that

$$f(v_t) \quad = \quad x_{t+1} + \epsilon_{t+1} \tag{2}$$

where ϵ_{t+1} is independent and identically distributed noise (IID). Although IID is sufficient tonsure that the model f captures the "interesting" dynamics and that the residuals ϵ are unbiased, we will later restrict this to being Gaussian as a computational expediency — again, not something that is central to the current document.

Note that this background with a problem in nonlinear time series analysis has now led us to a point where we wish to obtained a scalar function of a vector variable — a fairly standard problem in interpolation or surface fitting. Nonetheless, in the next section we present the particular approach we have chosen to take.

2 Radial Basis Functions and Minimum Description Length

The function f is essential a surface fit intended to interpolate a sequence of observed data pairs $(v_t; x_{t+1})$. Functional approximation tells us [1] that among the many possibilities, radial basis functions offer a good choice with compact support, infinite differentiability, and relatively straightforward minimisations. Moreover, these functions are mathematically well understood and have an established pedigree [3].

We approximate the function f with a some of radial basis terms, with the addition of linear dependence (capturing potential autoregressive processes in the time series problems, and linear correlation in the more general setting). In what follows we maintain the indexing by t, but this does now not necessarily represent time sequencing of points and the observed data pairs $(v_t; x_{t+1})$ may

[1] There are complications and extensions of this scheme which will provide improved results: selection of embedding dimension and *embedding lag* as well as non-uniform and even variable embedding strategies. However none of this machinery is necessary for the current discussion.

[2] If the topological space is "flat enough" — again, something that does not concern us here.

be more generic. The radial basis function takes the form:

$$f(x_t, x_{t-1}, \ldots, x_{t-d+1}) \quad = \quad \sum_{i=1}^{m} \lambda_i x_{t-\ell_i} + \sum_{i=m}^{M} \lambda_i \phi \left(\frac{\|v_t - c_i\|}{r_i} \right) \tag{3}$$

where $\ell_i > \ell_{i-1}$ are an increasing sequence of lags selecting some subset of the past values which are significant. The function $\phi : \mathbb{R}^d - \to \mathbb{R}$ defines the shape of the basis functions (we'll mainly use Gaussians) and the parameters $c_i \in \mathbb{R}^d$ and $r_i \in \mathbb{R}$ are the centre and radius of each basis function. Note that the model is linear in the parameters λ_i (hence this model formulation is referred to as *pseudo-linear* [2]), but the dependence on parameters ℓ_i, c_i and r_i may be highly nonlinear.

Before considering the selection of these various model parameters in more detail is is important to reflect on the most significant parameter of all — M, the model size. Given that the observed data set is (almost always) finite, if M is sufficiently large then the model can be fit arbitrarily well. However, this is *over-fitting* and will not lead to good generalisation or good performance of the model. Conversely, if M is too small the model will not contain essential dynamical features and the errors will include this structure as bias. A compromise must be found.

One (rather common) approach to this conflict is to separate the observed data to a *training* (or "fitting") and *testing* set. The model is fit only on the training data and the model parameters are tuned to that. The process is repeated for different choice of model size M and one chooses the model that performs best on the otherwise unseen testing data. The disadvantage of this approach is that only half the data is used to build the actual model. We employ a computational alternative to this heuristic. Description length is described in detail in the book of Rissanen [4], we provide only a brief *precise*.

Roughly, description length a measure of the compression achieved by describing a model and model prediction errors rather than describing the original data. If a model is a good model, then it provides a compact description of the data and the unknown data values can easily and cheaply be recovered by applying the model to the known input data. However, if a model is too large and consequentially a bad model, then the advantage of describing the data through the model prediction error is out weighted by the cost of describing the complexities of the model. Hence, as a function of M description length consists of two components: (1) the cost of describing the model, which increases with increasing M; and, (2) the cost of recovering the data through the model prediction errors, which decreases with M. The optimal value of M occurs when these two costs balance and we achieve a minimum. The *minimum description length principle* states that this value of M provides the best model, given the data.

Computationally, description length $L(x, \theta)$ can be computed as follows. As above x is the data and y the correspond n unknowns and we use θ to represent a vector of all the model parameters.

Let V be the $n \times M$ matrix which is the evaluation of each basis function

on each data point I.e. $V_{ij} = \phi_j(x_{i-1+d}, \ldots, x_i)$. The model (3) can then be expressed in vector notation as

$$e = y - V\lambda \tag{4}$$

where y and e are the corresponding unknowns and model error vectors (length n).

Assume that the errors e have a Gaussian distribution $e \sim N(0, \sigma^2)$ and hence their likelihood $P_e = \frac{1}{\sqrt{2\pi\sigma^2}} e^{-\frac{e^2}{2\sigma^2}}$. The description length of the data and the parameters is then expressed in two terms, the first the description length of the errors (the negative log-likelihood) and the second the description length of the parameters:

$$\begin{aligned} L(y,\theta) &= -\sum_{t=1}^{n} \log_2(P_{e_t}) + L(\theta) \\ &= \frac{n}{2}\log_2 2\pi\sigma^2 + \frac{1}{2\sigma^2}(y - V\lambda)^t(y - V\lambda) + L(\theta) \end{aligned}$$

We seek the best model, and hence the optimal values of $\lambda = \hat{\lambda}$ and σ: $D_\lambda L(x,\theta) = 0$:

$$\begin{aligned} -\frac{1}{\sigma^2} V^T(y - V\lambda) &= 0 \\ \hat{\lambda} &= (V^T V)^{-1} V^T y \end{aligned}$$

$D_{\sigma^2} L = 0$:

$$\begin{aligned} \frac{n}{2}\frac{1}{\sigma^2} - \frac{1}{2(\sigma^2)^2}(y - V\lambda)^T(y - V\lambda) &= 0 \\ \sigma^2 &= (y - V\lambda)^T(y - V\lambda)\frac{1}{n} \\ &= \frac{e^T e}{n} \end{aligned}$$

Let δ be the precision of λ and η be the precision of σ^2 (yes, we need that too).

$$\begin{aligned} Q &= \begin{bmatrix} D_{\lambda\lambda} L(y,\theta) & D_{\lambda\sigma^2} L(y,\theta) \\ D_{\sigma^2\lambda} L(y,\theta) & D_{\sigma^2\sigma^2} L(y,\theta) \end{bmatrix} \\ D_{\lambda\lambda} L(y,\theta) &= -\frac{1}{\sigma^2} V^T V \\ D_{\lambda\sigma^2} L(y,\theta) &= (D_{\sigma\lambda^2} L(y,\theta))^T \\ &= -\frac{1}{(\sigma^2)^2} V^T(y - V\lambda) \\ D_{\sigma^2\sigma^2} L(y,\theta) &= \frac{n}{2}\frac{1}{(\sigma^2)^2} \end{aligned}$$

But $D_\lambda L(y,\theta)=0$ means that $V^T(y-V\lambda)=0$, and hence

$$Q = \begin{bmatrix} -\frac{1}{\sigma^2}V^TV & 0 \\ 0 & -\frac{n}{2}\frac{1}{(\sigma^2)^2} \end{bmatrix}$$

which implies δ and η can be obtained as follows:

$$\left[\frac{1}{\sigma^2}V^TV\delta\right]_j = \frac{1}{\delta_j} \tag{5}$$

$$\frac{n}{2}\frac{1}{(\sigma^2)^2}\eta = \frac{1}{\eta}$$

$$\eta = \sqrt{\frac{2}{n}}\sigma^2 \tag{6}$$

Finally:

$$\begin{aligned} L(y,\theta) &= \frac{n}{2}\log_2\left(2\pi\frac{e^Te}{n}\right) + \frac{1}{2\sigma^2}e^Te - \sum_{j=1}^{k}\log_2\delta_j + \log_2\left(\sqrt{\frac{n}{2}}\frac{1}{\sigma^2}\right) \\ &= \frac{n}{2}\log_2 2\pi\frac{e^Te}{n} + \frac{n}{2} - \sum_{j=1}^{n}\log_2\delta_j + \log_2\left(\sqrt{\frac{2}{n}}\frac{e^Te}{n}\right) \\ &= \underbrace{\frac{n}{2}\log_2 2\pi + \frac{n}{2} - \frac{1}{2}\log_2\frac{n}{2}}_{\text{mostly constant}} + \underbrace{(\frac{n}{2}+1)\log_2\frac{e^Te}{n}}_{\propto\ L(x|\theta)=L(e)} - \underbrace{\sum_{j=1}^{k}\log_2\delta_j}_{\propto\ L(\theta)} \end{aligned}$$

Although messy, this last expression is entirely computable. The first part is largely constant and not required for optimisation of model size M, the second part is the cost of the model prediction errors, and the final term the cost of the model parameters expressed in terms of the precisions. The precisions, in turn, are computed as the solution of Eqn. (5).

Finally, we need to discuss the choice of the basis functions ϕ and the optimisation of the nonlinear parameters. We choose Gaussian basis $\phi(x) = \exp(-x^2/2)$ as we find that it works well in a wide variety of situations. This choice, however is arbitrary.

Nonetheless, the choice of Gaussians mean that the basis function are such that they have local effect centred about the parameter c_i (the centre). Away from c_i the contribution of each basis function vanishes. The rate at which that influence vanishes is dictated by r_i (the radius). Hence, both centre and radius can be chosen heuristically and optimised locally to place basis functions where the error is currently largest. Sensitivity analysis is then performed to select amongst an ensemble of potential basis functions and between those already in the existing model [2]. The linear parameters can be computed directly via ordinary least squares and matrix pseudo-inverse.

3 Adaption to animal behaviour

The modelling process described so far is intended to application to time series data. However, the extension to arbitrary input-output relationships, and hence the animal behaviour problems discussed in our paper is entirely straightforward. Removing the temporal dependence and modifying the penalty cost within the description length calculation, one can follow exactly the same procedure. For a known set of data x_i and corresponding unknown scalar targets y_i (formerly, this was $(x_t; y_{t+1})$, the change is only notational). The matrix V is formed from the evaluation of the basis functions on the data y, the errors e are the difference between the linear combination of these evaluations and the target data values y. Hence, following the procedure described above, one achieves a deterministic causal relationship $f(x) \approx y$ from which the main results of this paper stem.

Acknowledgements

MS is currently funded by the Australian Research Council via a Future Fellowship (FT110100896) and Discovery Project (DP140100203).

References

[1] E.W. Cheney. *Introduction to Approximation Theory.* American Mathematical Society, Providence, Rhode Island, 2 edition, 1982.

[2] Kevin Judd and Alistair Mees. On selecting models for nonlinear time series. *Physica D*, 82:426–444, 1995.

[3] M. J. D. Powell. The theory of radial basis function approximation in 1990. In Will Light, editor, *Advances in Numerical Analysis. Volume II: wavelets, subdivision algorithms and radial basis functions*, chapter 3, pages 105–210. Oxford Science Publications, 1992.

[4] Jorma Rissanen. *Stochastic complexity in statistical inquiry.* World Scientific, Singapore, 1989.

[5] Michael Small. *Applied Nonlinear Time Series Analysis: Applications in Physics, Physiology and Finance*, volume 52 of *Nonlinear Science Series A*. World Scientific, Singapore, 2005.

[6] Floris Takens. Detecting strange attractors in turbulence. *Lecture Notes in Mathematics*, 898:366–381, 1981.
